# Novel acute hypersensitivity pneumonitis model induced by airway mycosis and high dose lipopolysaccharide

**DOI:** 10.1186/s12931-021-01850-5

**Published:** 2021-10-10

**Authors:** Yuying Zeng, Yun Zhang, Xinyan Huang, Lizhen Song, Katherine Polsky, Yifan Wu, Farrah Kheradmand, Yubiao Guo, Linda K. Green, David B. Corry, John M. Knight

**Affiliations:** 1 Division of Pulmonary and Critical Care Medicine, The First Affiliated Hospital of Sun Yat-Sen University, Guangzhou, 510080 China; 2 Department of Medicine, Biology of Inflammation Center, Baylor College of Medicine, One Baylor Plaza, Houston, TX 77030 USA; 3 Department of Pathology & Immunology, Biology of Inflammation Center, Baylor College of Medicine, One Baylor Plaza, Houston, TX 77030 USA; 4 Biology of Inflammation Center, Baylor College of Medicine, One Baylor Plaza, Houston, TX 77030 USA; 5 Michael E. DeBakey VA Center for Translational Research on Inflammatory Diseases, Houston, TX 77030 USA; 6 Department of Pathology and Immunology, Michael E. DeBakey VA Center, 2002 Holcombe Boulevard, Houston, TX 77030 USA

**Keywords:** Acute hypersensitivity pneumonitis, Fungi, LPS, T_H_1, T_H_2, T_H_17 response

## Abstract

**Background:**

Inhalation of fungal spores is a strong risk factor for severe asthma and experimentally leads to development of airway mycosis and asthma-like disease in mice. However, in addition to fungal spores, humans are simultaneously exposed to other inflammatory agents such as lipopolysaccharide (LPS), with uncertain relevance to disease expression. To determine how high dose inhalation of LPS influences the expression of allergic airway disease induced by the allergenic mold *Aspergillus niger* (*A. niger*).

**Methods:**

C57BL/6J mice were intranasally challenged with the viable spores of *A. niger* with and without 1 μg of LPS over two weeks. Changes in airway hyperreactivity, airway and lung inflammatory cell recruitment, antigen-specific immunoglobulins, and histopathology were determined.

**Results:**

In comparison to mice challenged only with *A. niger*, addition of LPS (1 μg) to *A. niger* abrogated airway hyperresponsiveness and strongly attenuated airway eosinophilia, PAS+ goblet cells and T_H_2 responses while enhancing T_H_1 and T_H_17 cell recruitment to lung. Addition of LPS resulted in more severe, diffuse lung inflammation with scattered, loosely-formed parenchymal granulomas, but failed to alter fungus-induced IgE and IgG antibodies.

**Conclusions:**

In contrast to the strongly allergic lung phenotype induced by fungal spores alone, addition of a relatively high dose of LPS abrogates asthma-like features, replacing them with a phenotype more consistent with acute hypersensitivity pneumonitis (HP). These findings extend the already established link between airway mycosis and asthma to HP and describe a robust model for further dissecting the pathophysiology of HP.

**Supplementary Information:**

The online version contains supplementary material available at 10.1186/s12931-021-01850-5.

## Background

Asthma, including both allergic and non-allergic forms, is one of the most common chronic disorders affecting both children and adults. Allergic or atopic asthma, also termed extrinsic or T2 high asthma, is the most common form, characterized by a predominant T_H_2 cell immune response hallmarked by the production of the cytokines interleukin 4 (IL-4) and IL-5, and IL-13, eosinophilic inflammation, airway hyperreactivity, and increased serum IgE levels [1, 2]. Although immediate-type hypersensitivity to environmental antigens (atopy) is both a risk factor for and part of the pathophysiology of allergic asthma, exposure to fungi leading to airway mycosis, a form of non-invasive airway fungal infection, is also essential to disease expression [3–5]. In contrast, non-allergic asthma, also termed intrinsic or T2 low asthma, is not linked to atopy or eosinophilia, but instead to enhanced Th17 responses and neutrophilia while retaining airway hyperreactivity. Although airway mycosis has not been formally demonstrated in non-atopic asthma, fungal exposure is a strong risk factor for non-allergic asthma [6].

In addition to fungi, other environmental exposures, including lipopolysaccharide (LPS), appear to critically influence the expression of asthma. Humans with asthma concomitantly exposed to household LPS show reduced allergic sensitization [7]. Similarly, children raised in some farms appear to be protected from asthma and atopy, a beneficial effect that was traced to LPS exposure [8–11]. In contrast, exposure to some farm environments has been linked to higher asthma prevalence [12] and experimentally, LPS has been shown to promote the expression of allergic airway disease, a model of asthma, in mice [13].

Molecular elucidation of the complex relationship between LPS exposure and expression of allergic airway disease has been elusive, but evidence thus far indicates that LPS dose critically influences allergic outcomes, with higher LPS doses corresponding with protection and lower doses potentially exacerbating allergic inflammation. In this study, we examined the effect of high dose of LPS (1 µg) on airway mycosis-induced allergic airway disease in mice; in a parallel manuscript, we explore the effect of lower doses (10–100 ng) of LPS in the same model (Zeng et al. submitted). We demonstrate herein that the combined effect of fungal challenge with high dose LPS abrogates key features of asthma and instead results in a pattern of inflammation and lung histology that more closely resembles hypersensitivity pneumonitis (HP; extrinsic allergic alveolitis). Our findings highlight the complex effect that multiple airway exposures have on lung inflammation and the expanding role of airway mycosis in diverse lung disease contexts.

## Methods

### Mice

The C57BL/6J mice were purchased from The Jackson Laboratory (Bar Harbor, ME) and maintained at Baylor College of Medicine under specific pathogen-free (SPF) conditions. The female mice used were 5–8 weeks of age at the start of each experiment. All experimental protocols were approved by the Institutional Animal Care and Use Committee of Baylor College of Medicine and followed federal guidelines.

### Intranasal (in) challenge

**Fig. 1:**
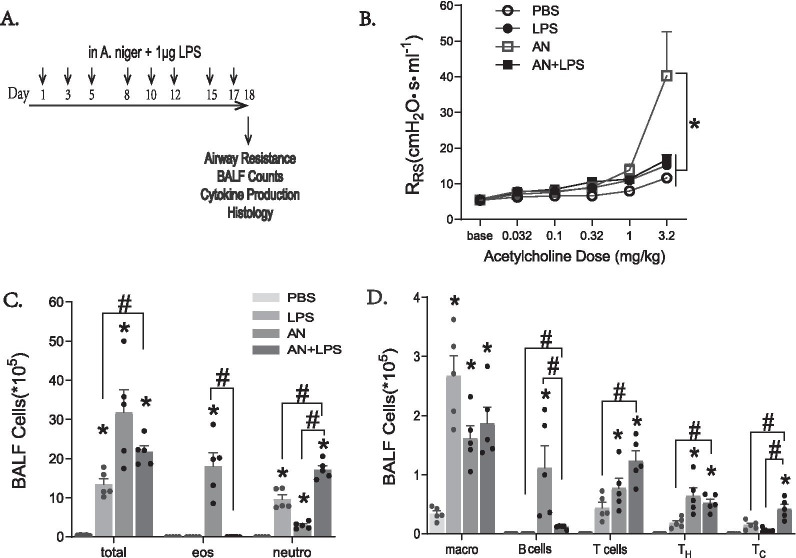
LPS attenuates fungal-induced asthma to promote neutrophilia (**A**) Mice were challenged intranasally with 4 × 10^5^ *Aspergillus niger* (AN) conidia and/or 1 μg LPS for 3 weeks as diagramed. The effects on airway hyperreactivity (**B**) and inflammatory cells in bronchoalveolar lavage fluid (BALF) (**C**, **D**) were determined. Results are presented as the mean ± SEM (n ≥ 3 in each group). *p < 0.05 compared with PBS administration; ^#^p < 0.05 between the indicated groups. Data are from one of two representative and independent experiments

The mice were randomly sorted into groups of 3–5 mice as needed for the experiment and challenged with 50 µL of PBS (Sigma-Aldrich) or viable *Aspergillus niger spores* (4 × 10^5^ spores) in the presence or absence of 1 μg LPS (*Esherichia coli* O127:B8, Sigma) 3 times per week for a total of 8 challenges, allowed to rest, and euthanized the following day as described in the Fig. 1A.

### Airway physiology

Bronchial responsiveness was determined by assessing the increase in respiratory system resistance (R_RS_) to 5 increasing intravenous acetylcholine doses, measured by whole body, semi-invasive plethysmography as previously described [14].

### Sample collection and single cell suspensions

Mice were intubated with a 20G ½ catheter (Smith Medical) and lavaged with 1.6 mL PBS. Cells were isolated from bronchoalveolar lavage fluid (BALF) by centrifugation and supernatant stored at − 80 ℃ for cytokine analysis. Lung homogenates were prepared from PBS perfused lungs by incubating mechanically processed lung tissue in digestion buffer with 2 mg/mL collagenase type II (Worthington), 0.04 mg/mL DNase I (Sigma-Aldrich), 20% FBS (Gibco) in 1X HBSS (Gibco) for 30 min at 37 ℃ and passed through a 70um mesh basket (Fisher Scientific). Lung homogenates were further processed for flow cytometric analysis or cultured in complete RPMI 1640 medium (GenClone) at 37 ℃ with 5% CO_2_ overnight. Lung cell culture supernatants were collected the next day and stored at − 80℃ for cytokine analysis by multiplex.

### Cytokine and chemokine analysis

The indicated cytokines and chemokines were measured in BALF or lung supernatant by using mouse cytokine/chemokine magnetic bead and mouse Th17 magnetic bead panel kits (Millipore) on a Luminex-based multianalyte platform (Bio-Plex; Bio-Rad, Hercules, CA) following the manufacture’s recommended procedures.

### Flow cytometric analysis

For flow cytometric analysis of inflammatory cells, single-cell suspensions of BALF cells or lung single-cell suspensions were preincubated with a blocking cocktail of anti-mouse CD16/32 (clone 93, Biolegend) for 10 min at 4 ℃. Differential staining of inflammatory cells recruited to the lungs, cells were labeled with CD11b-PE (clone M1/70, Invitrogen), CD11c-APC (clone HL3, BD Pharmingen), Siglec-F-PerCP-Cy5.5 (clone E50-2440, BD Pharminge), Ly6G/C-APC-Cy7 (clone HL3, BD Pharmingen), CD3-eFluor450 (clone 17A2, ThermoFisher), CD19-BV510 (clone 6D5, Biolegend), CD4-BV785 (clone RM4-5, Biolegend), CD8a-BV711 (clone 53–6.7, Biolegend) and Fixable Blue dead cell stain (ThermoFisher) in filtered FACS buffer containing 1% BSA (GenDEPOT) and 0.4% 0.5 M EDTA (Gibco). Then lysing solution (BD Biosciences) was used to lyse red blood cells. For the intracellular cytokine staining, disaggregated lung cells were stimulated with 50 ng/mL PMA (Biogems) and 500 ng/mL ionomycin (Biogems) in the presence of brefeldin A (Biolegend) for 4 h at 37 ℃. Surface markers and viability were stained (CD4-APC (clone RM4-5, Biolegend), CD3ε-APC-Cy7 (clone 145-2C11, Biolegend), Fixable Blue dead cell stain), following by red blood cell lysed, permeabilized and intracellular cytokines IFN-γ-BV750 (clone XMG1.2, BD Bioscience), IL-5-PE (clone TRFK5, BD Pharmingen), IL-13-Alexa Fluor 488 (clone eBio13A, ThermoFisher) and IL-17A-BV650 (clone TC11-18H10.1, Biolegend) were stained in accordance to the protocol of Cytofix/Cytoperm Plus Kit (BD Biosciences). Finally, the labeled cells were acquired on a BD LSRFortessa cytometry (BD Biosciences) and analyzed by using FlowJo 10.4 software (Ashland, OR). Gates were determined by comparison of FMO and fully stained samples, and absolute cell numbers were quantified by addition of precision count beads (Biolegend) in accordance to the manufactures protocol.

### Fungal antigen preparation

Fungal antigen was prepared as previously described [5]. Briefly, hyphae mats were collected from conidia incubating in autoclaved Sabouraud broth (BD Difco) and ground colloidally in a planetary ball mill (Retsch/Verder Scientific, Newtown, Pa) with 2-mm zirconium oxide balls in PBS. After centrifuge, the supernatant was passed through a 0.22 μm filter (VWR) and protein concentration was determined by BCA protein assay kit (Thermo Scientific). Small aliquots were stored at − 80 ℃ until use.

### ELISA immunoglobulin assay

Immunoglobulins in serum were semi-quantified as optical density (O.D.) readings via sandwich ELISA. Briefly, sera were added to anti-mouse IgE capture antibody (BD Pharmingen) or fungal antigen coated ELISA-grade plate (Corning), followed by incubation with biotin IgE or IgG1 or IgG2a (BD Pharmingen) or HRP-conjugated IgG (Southern BioTech). Sav-HRP (BD Biosciences) was used to bind with HRP-unconjugated detection antibodies and the activity of HRP was detected by TMB substrates (Thermo Scientific).

### Histology

Lungs were inflated with 10% neutral-buffered formalin (Sigma-Aldrich) at 25 cm H_2_O. Paraffin-embedded sections (5um) were stained with hematoxylin and eosin (H&E), periodic acid-Schiff (PAS) (Thermo Scientific) or Masson’s Trichrome (Thermo Scientific) for histopathologic analysis according to the manufacturer’ s protocols. Two slides of whole fixed and stained lung (5 μg) were prepared for each mouse (n = 5 per group) and reviewed for pathologic changes and quantification of granulomas. Each slide contained the entire mouse lung separated into individual lobes and sectioned so as to capture peripheral and central lung.

### Statistics

Data were analyzed with GraphPad Prism 8 and presented as the mean ± SEM. Datasets were tested for normality and homogeneity of variances, and comparisons were made with one-way ANOVA with Dunnett’s test or Student’s unpaired *t* test or two-way ANOVA with Benjamineni, Krieger and Yekutieli post test. p < 0.05 was considered statistically significant.

## Results

### High dose LPS attenuates key features of airway mycosis-induced allergic airway disease

Low doses of LPS have previously been shown to elicit allergic airway disease in mice, but less is known about the effect of higher LPS doses. To investigate this, we challenged mice intranasally with 1 µg LPS over two weeks with and without concomitant exposure to the viable spores of *A. niger* (Fig. 1A). As expected, challenge of mice with *A. niger*, which results in an active airway infection (airway mycosis), induced airway hyperreactivity as assessed by increases in respiratory system resistance (R_RS_) in response to intravenous acetylcholine challenge accompanied by predominant airway eosinophilia as compared to PBS challenged controls (Fig. 1B, C; Additional file 1: Fig. S1A) [1]. In contrast, the addition of LPS to *A. niger* spores abrogated airway hyperreactivity and eosinophilia, and instead induced a robust neutrophilia. LPS challenge alone failed to induce airway hyperreactivity, but also elicited a substantial neutrophilia (Fig. 1B, C).

Total inflammatory cells in bronchoalveolar lavage fluid (BALF) and lungs were significantly increased in all challenged mice when compared to naïve animals, but both LPS treated groups had significantly fewer cells in BALF as compared to the fungal challenge alone (Fig. 1C) although total lung inflammatory cells were preserved after addition of LPS to *A. niger* (Additional file 1: Fig. S1B). Macrophage abundance was increased in all treated groups relative to PBS, but analysis of lymphocyte populations showed marked recruitment of B cells only to the airways in fungal-challenged mice (Fig. 1D), a pattern that was largely matched by analysis of whole lung (Additional file 1: Fig. S1B). Mice challenged with fungus and LPS had significantly more T cells recruited to airways and lungs when compared to LPS or fungus alone, due to enhanced CD8+ T cell recruitment (Fig. 1D; Additional file 1: Fig. S1B). Thus, high-dose LPS challenge abrogates airway mycosis-induced airway hyperreactivity and eosinophilic inflammation, replacing these features of asthma with a neutrophilic inflammatory process with enhanced T cell recruitment.

### LPS suppresses T_H_2 and *A. niger*-induced CD4+ T helper cell recruitment to lung

**Fig. 2:**
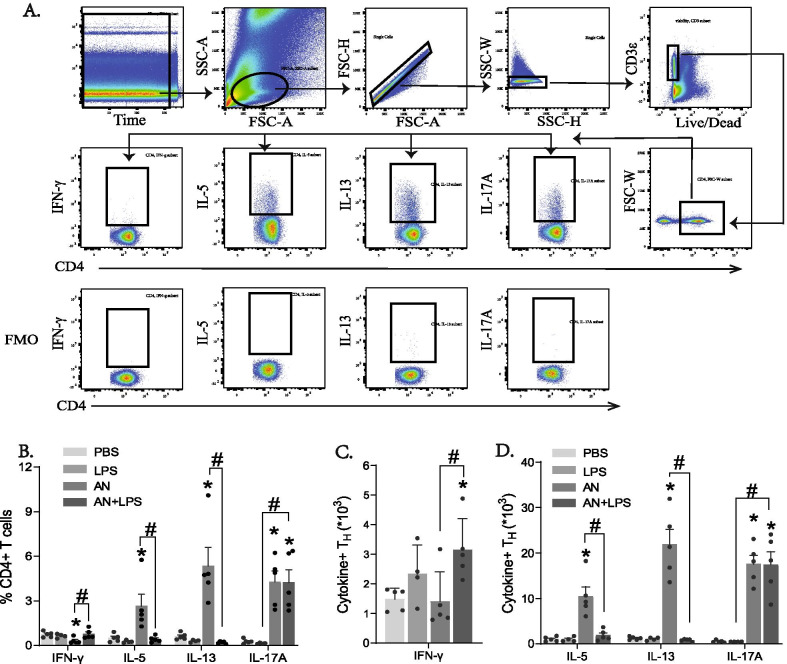
LPS attenuates T_H_2 inflammatory cytokine production by CD4+ T cells in lung. **A** Flow cytometric gating scheme on whole lung cells. **B** The percentage of different T helper cells in the lung. Total number of **C** IFN-γ^+^ and **D** IL-5, IL-13, and IL-17A positive CD3^+^ CD4^+^ cells in lungs. Results are presented as the mean ± SEM (n ≥ 4 in each group). *p < 0.05 compared with PBS administration. ^#^p < 0.05 between indicated groups. Data are from one of two representative and independent experiments

We conducted additional studies to determine how high dose LPS influences the recruitment of T effector cells to the lung using flow cytometry and intracellular cytokine staining (Fig. 2A). Expressed as a fraction of total CD4+ T cells, LPS challenge alone failed to elicit recruitment of any effector T cell, but challenge with *A. niger* resulted in the robust recruitment of IL-5 and IL-13 secreting cells, consistent with a substantial T_H_2 response, together with IL-17A-secreting T_H_17 cells (Fig. 2B, D). Intriguingly, addition of LPS to *A. niger* spores abrogated expression of T_H_2 cytokines without affecting the relative abundance of T_H_17 cells. Enumeration of lung T effector cells under the same challenge conditions confirmed these observations but extended them by revealing that addition of LPS to *A. niger* resulted in the significant recruitment of IFN-γ-secreting T_H_1 cells. (Fig. 2B, C). Notably however, even in the combined challenge group, T_H_1 cell abundance was far lower than other T effector cells (Fig. 2C vs D.).

**Fig. 3:**
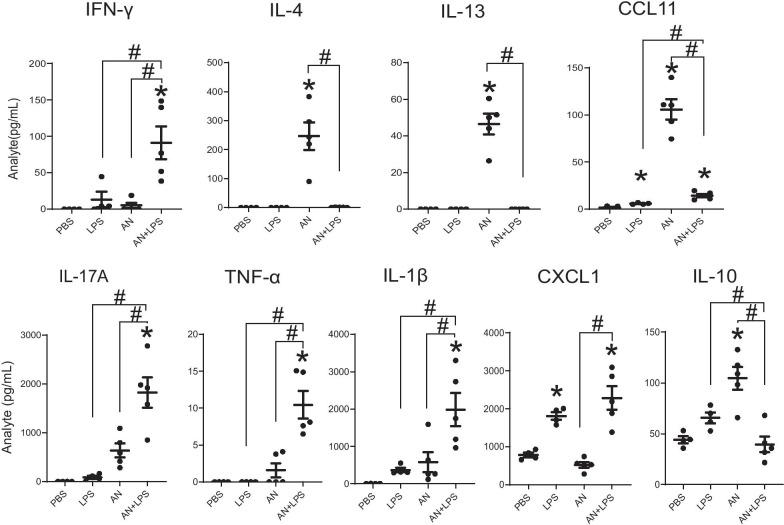
Effect of LPS on cytokines and chemokines in lung culture supernatants measured by Multiplex Luminex-based assay. Results are presented as the mean ± SEM (n ≥ 4 in each group). *p < 0.05 compared with PBS administration; ^#^p < 0.05 between the indicated groups. Data are from one of two representative and independent experiments

We further quantified cytokines secreted from deaggregated whole lung under the same challenge conditions (Fig. 3). The T_H_1 cytokine IFN-γ was again most highly secreted from lungs of fungus and LPS-challenged mice. Significant secretion of the T_H_2 cytokines IL-4 and IL-13 and the T_H_2-related chemokine CCL11 (eotaxin) was observed from lungs of *A. niger* challenged mice, but this was extinguished by addition of LPS. In contrast, the T_H_17-related cytokine IL-17A was secreted from lung to a significant degree only in mice challenged with both *A. niger* and LPS, a pattern that was matched by the pro-inflammatory cytokines TNF-α and IL-1β (Fig. 3). The enhanced effect of *A. niger* and LPS challenge on IL-17A secretion was not matched by equivalent increases in Th17 cells (Fig. 2D), suggesting the possible contribution of other IL-17A-secreting cells including γδ, ILC3, and T_C_17 cells. Secretion of the neutrophilia-inducing chemokine CXCL1 was significantly increased in supernatants from both mouse groups challenged with LPS, potentially accounting for the neutrophilia observed in the same groups (Fig. 1C). Similar to T_H_2 cytokines, the immunosuppressive cytokine IL-10 was secreted to a significant degree only in mice challenged with *A. niger*. Taken together, these findings confirm that high dose LPS potently suppresses airway mycosis-dependent lung T_H_2 immune responses in the lungs that drive asthma development.

### Fungus-specific immunoglobulin secretion is unaffected by LPS

**Fig. 4:**
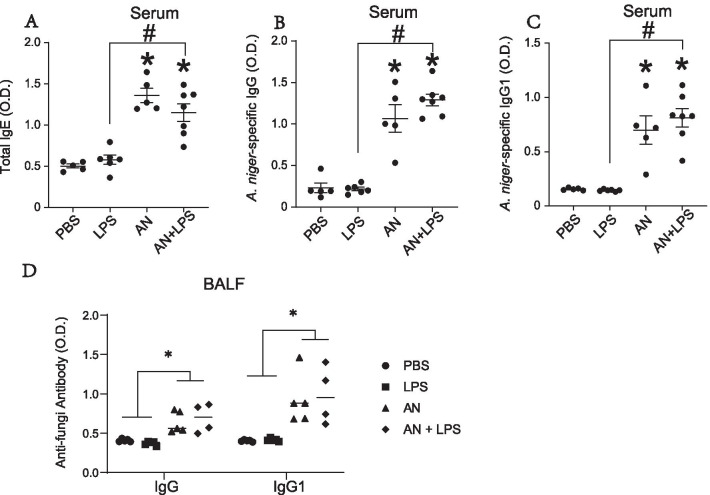
The serum and BALF antibody levels were detected by ELISA. **A** Total IgE; **B** Anti-fungi IgG; **C** Anti-fungi IgG1. **D** BALF Anti-fungal antibody. Results are presented as the mean ± SEM (n ≥ 5 in each group). *p < 0.05 between indicated groups. Data are from one of three representative and independent experiments

LPS exposure powerfully modifies pulmonary immune responses, including systemic immunoglobulin secretion [15]. To address the potential of LPS to skew immunoglobulin production in our model, we quantified total and antigen specific serum antibodies. Circulating IgE was significantly increased in fungal challenged groups and was not impacted by LPS challenge (Fig. 4A). Antigen specific total IgG and fungus-specific IgG1 were also observed in both fungus challenge groups, but also not significantly affected by treatment with LPS (Fig. 4B, C). Fungus-specific IgG2a was undetectable in all groups (data not shown).

In addition to systemic antibody, airway antigen exposure leads to the accumulation of immunoglobulins within the airways [16], which can be markedly enhanced by exposure to LPS [17]. In contrast to these studies that did not involve fungal challenge, exposure of mice to inhaled LPS failed to augment the airway immunoglobulins already induced by fungal challenge (Fig. 4D). Thus, humoral responses to fungal infection are maintained in the serum and airway despite the phenotypic switch away from a dominant T_H_2 immune response induced by high dose LPS (Additional file 2: Table S1).

### The combination of *A. niger* and LPS elicits granulomatous lung inflammation

**Fig. 5:**
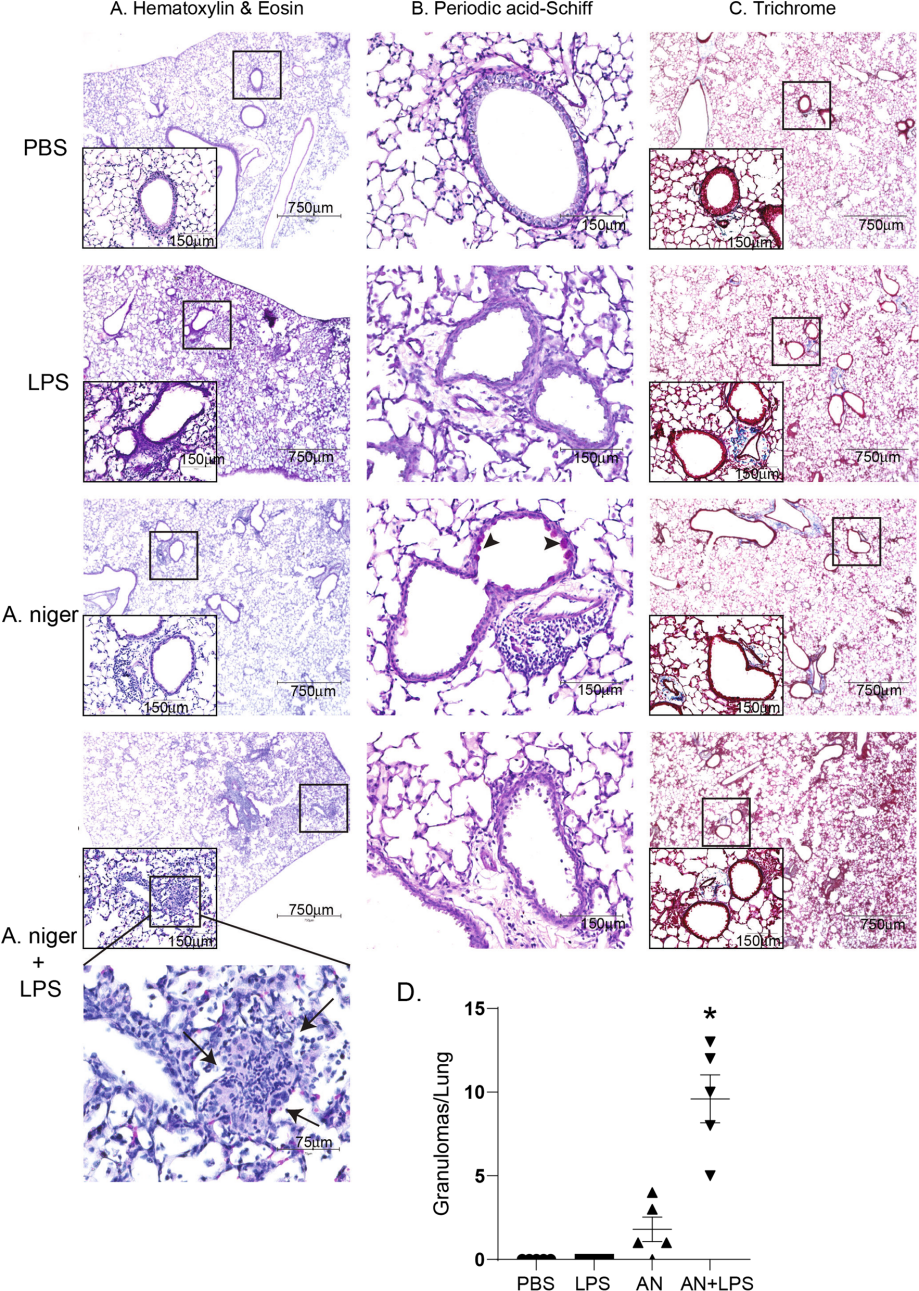
The effect of LPS on airway inflammation, mucus production and collagen deposition in lung tissue. **A** Hematoxylin and eosin (H&E) staining. Loosely-formed granulomas are indicated by arrows. **B** Periodic acid-Schiff (PAS) staining. Goblet cells are indicated by arrowheads. **C** Masson’s trichrome (Trichrome) staining. The scale bars in lowest magnification are 750 μm. The scale bars in higher magnification are 150 μm. The scale bar in highest magnification is 75 μm. **D** Quantification (mean ± SD) of loosely-formed granulomas from lungs of the different treatment groups. *p < 0.05 relative to all other groups. Data are from one of two representative and independent experiments

Finally, we conducted careful histologic analyses of mouse lung to understand the effect of high dose LPS addition to *A. niger* (Additional file 3: Table S2). Compared to PBS-challenged mice, all other challenged mice showed increased cellular infiltration and peri-bronchovascular bundle cellularity suggestive of the development of tertiary lymphoid tissue (TLT; Fig. 5A) [18]. Goblet cell metaplasia that was readily found in *A. niger*-challenged mice was absent in LPS-treated mice with airway mycosis (Fig. 5B). Trichrome staining failed to show significant fibrosis in any of the challenge groups (Fig. 5C). Unexpectedly, we identified (Fig. 5A) and quantified (Fig. 5D) multiple loosely-formed granulomas including occasional multinucleated giant cells admixed with lymphocytes and histiocytes primarily in the lung parenchyma of LPS and *A. niger* challenged mice, although some granulomas were found in mice challenged only with *A. niger* (Fig. 5D). Together, these histologic observations confirm that addition of high dose LPS to *A. niger* challenge abrogates canonical features of asthma. The histologic findings when viewed together with the immunological data overall support a disease pattern that most closely resembles hypersensitivity pneumonitis.

## Discussion

Using improved culture techniques, numerous common environmental fungi are readily identified from spontaneously produced human sputa [4, 19–21] and these fungi all have the potential to induce airway mycosis and asthma-like allergic airway disease in mice [1, 22]. These observations together with extensive epidemiologic [23–26], hypersensitivity [27–33], antifungal clinical trial [34, 35], and other studies [5, 36–41] support the relatively recent recognition that airway mycosis underlies severe, treatment-refractory forms of asthma [2]. However, in addition to fungal spores, humans routinely inhale other pro-inflammatory environmental agents such as LPS. To further understand the relationship between LPS exposure and airway mycosis in the pathogenesis of inflammatory airway disease, we have exposed mice to the combination of high-dose LPS and the viable spores of *A. niger* in comparison to mice challenged separately with these agents. We have shown that the combination of LPS and *A. niger* spores abrogates the typical T_H_2-biased eosinophilic inflammation that, together with T_H_17 cells, are highly characteristic of severe allergic asthma [42] and fungal-challenged mice with fungus-induced allergic airway disease [22]. We have further shown that high dose LPS challenge of mice with airway mycosis alters the peri-bronchovascular inflammation and goblet cell metaplasia typical of asthma to a more diffuse neutrophil and T_H_17-cell-predominant inflammatory infiltrate that includes occasional poorly formed pulmonary parenchymal granulomas. Combined with the preservation of IgE responses, the findings collectively yield a pattern of inflammation and lung injury that most closely resembles hypersensitivity pneumonitis (HP; extrinsic allergic alveolitis) and indicate that HP exists along a continuum of airway mycosis-induced diseases that include both asthma and HP.

HP is an interstitial inflammatory lung disease characterized by alveolitis, peribronchial granuloma formation and, in advanced stages, fibrosis [43]. The prevalence of HP is estimated to be 1–30 per 100,000, but the nonspecific clinical presentation and similarity in clinical findings to that of other common pulmonary inflammatory conditions makes estimations of disease occurrence difficult [44]. Certain subpopulations like farmers have a considerably higher occurrence (Farmer’s lung), presumably due to their occupational exposure to multiple environmental antigens. HP has been associated with more than 300 antigenic inorganic/chemical compounds, plant and animal proteins, and infectious bacterial and fungal pathogens [44]. Only a small percentage of antigen-exposed individuals will develop HP, however, suggesting that a genetic predisposition to disease may exist. HP can be subdivided into acute, subacute, and chronic forms depending on the severity and duration of disease, but the essential steps in the immunopathogenesis and progression of this disease are not fully understood [43].

The most common strategy to model HP acutely relies on repeatedly challenging mice with bacterial extracts from the thermophilic actinomycete *Saccharopolyspora rectivirgula* (SA; formerly *Micropolyspora faeni*), which is a hay-derived pathogen commonly associated with farmers lung [45–47]. *Aspergillus* species are also considered causative agents of Farmer’s lung and Mushrooms worker’s lung, two separately defined HP subtypes. The classification of different types of HP due to occupation highlights the disparity in studying what likely is a complex interaction of environmental and host interactions that result in an uncommon disease such as HP. It is likely that Farmer’s lung, mushroom worker’s lung, Hot tub lung and Breeder’s lung diseases are all considered HP due to markers of immune dysregulation that manifest similarly. Identification of the common inflammatory pathways of these different types of HP would shed light on the underlying disease mechanisms.

Early studies showed that T_H_1 immune responses promote HP through the combined effect of IFN-γ and IL-12 to promote neutrophil activation and recruitment to the lung [48]. Subsequent studies found that the T_H_1-defining transcription factor T-bet restrains T_H_17 immune responses to attenuate HP in mice [49]. More recent studies have shown an essential role for IL-17A and neutrophils in the development of peribronchiolar inflammation in acute HP [46, 50]. Although a role for both T_H_1 and T_H_17 immunity in the development of such inflammation is clear, *S. rectivirgula*-based antigen challenge models fail to elicit the pulmonary granulomas that typify acute HP, instead demonstrating the emergence of bronchus associated lymphoid tissue (BALT) and other TLT. Moreover, fungi such as *Aspergillus* spp. have also been epidemiologically and serologically linked to HP and a more complex immune response that includes T_H_2 cell activation in more severe and chronic forms of HP [51, 52]. Notably, inclusion of fungi and viable organisms of any type in models of HP has been lacking thus far.

Unexpectedly, we found that the combination of viable *A. niger* spores and high-dose LPS reproduces key features of acute HP including the lack of airway hyperreactivity and eosinophilia; substantial neutrophilia; enhanced CD8+ cytotoxic T cells recruitment; and reduced or absent T_H_2 and predominant T_H_1 and T_H_17 cytokines. Our model also replicates the poorly-formed granulomas seen in HP in addition to TLT, suggesting that the inclusion of viable fungi capable of inducing airway mycosis [1] may be critical to more accurately modeling HP. Inviable fungal spores are specifically immunologically inert due to their hydrophobin-coated exteriors [1, 53]. While formal diagnostic criteria for HP, including acute, subacute, and chronic forms are not widely agreed upon, the finding of poorly formed granulomas combined with neutrophilia and lack of fibrosis and eosinophils are most consistent with acute HP [52, 54, 55]. The relatively short period of fungal challenge (2 weeks) further supports a model of acute disease.

TLR4 agonists such as LPS and other pathogen associated molecular patterns (PAMPs) are powerful immune modifiers that can influence expression of HP-like disease in rodents. For example, LPS strongly augments airway IgG in mice challenged with *Methanosphaera stadtmanae* [17]. In a separate model of *Mycobacterium immunogenum*-induced pulmonary inflammation, addition of LPS augmented pulmonary pathology, including granulomatous changes [56]. Viruses such as influenza A induce pulmonary inflammation in part by signaling through TLR3 and related receptors and have been recovered from the lower airways of patients with HP, suggesting a pathophysiological link [57]. Consistent with this concept, experimental models demonstrated enhanced features of HP when viral infection was superimposed on the standard antigenic challenge with non-viable *Saccharopolyspora rectivirgula* [58, 59]. Although LPS profoundly altered the inflammatory and pathologic character of the mouse lung during viable fungal challenge, it did not alter pulmonary or systemic antibodies, further underscoring the unique nature of this new HP model.

Of note, the differential diagnosis of pulmonary syndromes involving well-developed granulomas can be challenging, especially when sarcoidosis is a consideration [60]. Our new model of acute HP is not a suitable model for sarcoidosis as this idiopathic disorder cannot be diagnosed as such in the context of a known infection. Sarcoidosis is furthermore characterized by the presence of innumerable well-formed granulomas, whereas our model demonstrates only uncommon, poorly-formed granulomas. It is the uncommon and poorly formed nature of the granulomas seen in this model that distinguish it from models of most other granulomatous disorders, especially sarcoidosis.

Subacute HP is characterized by the evolution of neutrophilic inflammation into a predominant lymphocytic infiltrate and the beginning of pulmonary fibrosis and reduced pulmonary function. The final, or chronic, stage of HP is marked by advanced pulmonary fibrosis, profound pulmonary ventilatory restriction, and hypoxemia that can be fatal. Removal from exposure to offending antigens is often, but not always, successful in ameliorating disease. Our findings suggest for the first time that, much like asthma, progressive, unremitting HP could be due to unresolved airway mycosis, but combined with either concomitant bacterial bronchitis or exposure to high dose LPS [or similar pathogen associated molecular pattern (PAMP)]. Therefore, in any patient suspected of having HP, our studies suggest that an aggressive search for airway mycosis be conducted, including assessment of anti-fungal antibodies and culture of airway samples, especially sputum or bronchoalveolar lavage fluid. Unfortunately, as we and others have previously published [5, 21], standard airway specimen fungal culture methods are inadequate, usually yielding no organisms with perhaps the exception of *Candida *spp., although enhanced sputum culture methods have been described [4]. Although controlled clinical trials are needed, persistent evidence of fungal airway infection should prompt aggressive antifungal management of the airway mycosis, which could potentially be lifesaving if found in especially refractory subacute and chronic HP. Future studies should therefore focus on establishing more clearly a link between HP and airway mycosis and expanding the currently described model to include features of subacute and chronic HP.

## Supplementary Information

**Additional file 1: Fig. 1:** The effect of LPS on the composition of inflammatory cells in the lungs. (A) Flow cytometric analytical scheme of the differential of BALF cells and lung cells. (B) Lung cells were analyzed for inflammatory cell numbers. Results are presented as the mean ± SEM (n=5 in each group). *p<0.05 compared with PBS administration; #p<0.05 between indicated groups. Data are from one experiment.

**Additional file 2: Table S1.** mouse antibody marker, fluorphore, clone and company used for cytometric analysis of lung cells.

**Additional file 3: Table S2.** Histopathologic description and score of mouse lungs by group..

## Data Availability

All source date pertinent to this study are stored on local servers and will be made available upon reasonable request without requiring an MTA.

## Abbreviations

BALF: Bronchoalveolar lavage fluid
BALT: Bronchus associated lymphoid tissue
HP: Hypersensitivity pneumonitis
LPS: Lipopolysaccharide

## References

[1] Porter P, Susarla SC, Polikepahad S, Qian Y, Hampton J, Kiss A, Vaidya S, Sur S, Ongeri V, Yang T (2009). Link between allergic asthma and airway mucosal infection suggested by proteinase-secreting household fungi. Mucosal Immunol.

[2] Li E, Knight JM, Wu Y, Luong A, Rodriguez A, Kheradmand F, Corry DB (2019). Airway mycosis in allergic airway disease. Adv Immunol.

[3] Porter P, Polikepahad S, Qian Y, Knight JM, Lu W, Tai WM, Roberts L, Ongeri V, Yang T, Seryshev A (2011). Respiratory tract allergic disease and atopy: experimental evidence for a fungal infectious etiology. Med Mycol.

[4] Mak G, Porter PC, Bandi V, Kheradmand F, Corry DB (2013). Tracheobronchial mycosis in a retrospective case-series study of five status asthmaticus patients. Clin Immunol.

[5] Porter PC, Lim DJ, Maskatia ZK, Mak G, Tsai CL, Citardi MJ, Fakhri S, Shaw JL, Fothergil A, Kheradmand F (2014). Airway surface mycosis in chronic TH2-associated airway disease. J Allergy Clin Immunol.

[6] Dannemiller KC, Gent JF, Leaderer BP, Peccia J (2016). Indoor microbial communities: influence on asthma severity in atopic and nonatopic children. J Allergy Clin Immunol.

[7] Gehring U, Bischof W, Fahlbusch B, Wichmann HE, Heinrich J (2002). House dust endotoxin and allergic sensitization in children. Am J Respir Crit Care Med.

[8] Braun-Fahrlander C, Riedler J, Herz U, Eder W, Waser M, Grize L, Maisch S, Carr D, Gerlach F, Bufe A (2002). Environmental exposure to endotoxin and its relation to asthma in school-age children. N Engl J Med.

[9] Feng M, Yang Z, Pan L, Lai X, Xian M, Huang X, Chen Y, Schroder PC, Roponen M, Schaub B (2016). Associations of early life exposures and environmental factors with asthma among children in rural and urban areas of Guangdong, China. Chest.

[10] Stein MM, Hrusch CL, Gozdz J, Igartua C, Pivniouk V, Murray SE, Ledford JG, Marques dos Santos M, Anderson RL, Metwali N (2016). Innate immunity and asthma risk in amish and hutterite farm children. N Engl J Med.

[11] Schuijs MJ, Willart MA, Vergote K, Gras D, Deswarte K, Ege MJ, Madeira FB, Beyaert R, van Loo G, Bracher F (2015). Farm dust and endotoxin protect against allergy through A20 induction in lung epithelial cells. Science.

[12] Wickens K, Lane JM, Fitzharris P, Siebers R, Riley G, Douwes J, Smith T, Crane J (2002). Farm residence and exposures and the risk of allergic diseases in New Zealand children. Allergy.

[13] Eisenbarth SC, Piggott DA, Huleatt JW, Visintin I, Herrick CA, Bottomly K (2002). Lipopolysaccharide-enhanced, toll-like receptor 4-dependent T helper cell type 2 responses to inhaled antigen. J Exp Med.

[14] Polikepahad S, Barranco WT, Porter P, Anderson B, Kheradmand F, Corry DB (2010). A reversible, non-invasive method for airway resistance measurements and bronchoalveolar lavage fluid sampling in mice. J Vis Exp.

[15] Wan GH, Li CS, Lin RH (2000). Airborne endotoxin exposure and the development of airway antigen-specific allergic responses. Clin Exp Allergy.

[16] Galvin JB, Bice DE, Muggenburg BA (1986). Comparison of cell-mediated and humoral immunity in the dog lung after localized lung immunization. J Leukoc Biol.

[17] Huppe CA, Blais-Lecours P, Bernatchez E, Lauzon-Joset JF, Duchaine C, Rosen H, Dion G, McNagny KM, Blanchet MR, Morissette MC, Marsolais D (2020). S1P_1_ contributes to endotoxin-enhanced B-cell functions involved in hypersensitivity pneumonitis. Am J Respir Cell Mol Biol.

[18] Huppe CA, Blais Lecours P, Lechasseur A, Gendron DR, Lemay AM, Bissonnette EY, Blanchet MR, Duchaine C, Morissette MC, Rosen H, Marsolais D (2018). A sphingosine-1-phosphate receptor 1 agonist inhibits tertiary lymphoid tissue reactivation and hypersensitivity in the lung. Mucosal Immunol.

[19] Li E, Tsai C-L, Maskatia ZK, Kakkar E, Porter P, Rossen RD, Perusich S, Knight JM, Kheradmand F, Corry DB (2018). Benefits of antifungal therapy in asthma patients with airway mycosis: a retrospective cohort analysis. Immun Inflamm Dis.

[20] Agbetile J, Fairs A, Desai D, Hargadon B, Bourne M, Mutalithas K, Edwards R, Morley JP, Monteiro WR, Kulkarni NS (2012). Isolation of filamentous fungi from sputum in asthma is associated with reduced post-bronchodilator FEV1. Clin Exp Allergy.

[21] Pashley CH, Fairs A, Morley JP, Tailor S, Agbetile J, Bafadhel M, Brightling CE, Wardlaw AJ (2012). Routine processing procedures for isolating filamentous fungi from respiratory sputum samples may underestimate fungal prevalence. Med Mycol.

[22] Porter P, Qian Y, Abramson S, Delclos GL, Kheradmand F, Corry DB (2011). Necessary and sufficient role for T helper cells to prevent fungal dissemination during mucosal airway infection. Infect Immun.

[23] Salo PM, Arbes SJ, Sever M, Jaramillo R, Cohn RD, London SJ, Zeldin DC (2006). Exposure to Alternaria alternata in US homes is associated with asthma symptoms. J Allergy Clin Immunol.

[24] Lyons TW, Wakefield DB, Cloutier MM (2011). Mold and Alternaria skin test reactivity and asthma in children in Connecticut. Ann Allergy Asthma Immunol.

[25] Quansah R, Jaakkola MS, Hugg TT, Heikkinen SA, Jaakkola JJ (2012). Residential dampness and molds and the risk of developing asthma: a systematic review and meta-analysis. PLoS ONE.

[26] Reponen T, Lockey J, Bernstein DI, Vesper SJ, Levin L, Khurana Hershey GK, Zheng S, Ryan P, Grinshpun SA, Villareal M, Lemasters G (2012). Infant origins of childhood asthma associated with specific molds. J Allergy Clin Immunol.

[27] Denning DW, O'Driscoll BR, Hogaboam CM, Bowyer P, Niven RM (2006). The link between fungi and severe asthma: a summary of the evidence. Eur Respir J.

[28] Niedoszytko M, Chelminska M, Jassem E, Czestochowska E (2007). Association between sensitization to *Aureobasidium pullulans* (*Pullularia* sp.) and severity of asthma. Ann Allergy Asthma Immunol.

[29] Pulimood TB, Corden JM, Bryden C, Sharples L, Nasser SM (2007). Epidemic asthma and the role of the fungal mold Alternaria alternata. J Allergy Clin Immunol.

[30] Farrant J, Brice H, Fowler S, Niven R (2016). Fungal sensitisation in severe asthma is associated with the identification of *Aspergillus fumigatus* in sputum. J Asthma.

[31] Segura N, Fraj J, Cubero JL, Sobrevia MT, Lezaun A, Ferrer L, Sebastian A, Colas C (2016). Mould and grass pollen allergy as risk factors for childhood asthma in Zaragoza, Spain. Allergol Immunopathol.

[32] Goh KJ, Yii ACA, Lapperre TS, Chan AK, Chew FT, Chotirmall SH, Koh MS (2017). Sensitization to *Aspergillus* species is associated with frequent exacerbations in severe asthma. J Asthma Allergy.

[33] Medrek SK, Kao CC, Yang DH, Hanania NA, Parulekar AD (2017). Fungal sensitization is associated with increased risk of life-threatening asthma. J Allergy Clin Immunol Pract.

[34] Ward GW, Woodfolk JA, Hayden ML, Jackson S, Platts-Mills TA (1999). Treatment of late-onset asthma with fluconazole. J Allergy Clin Immunol.

[35] Denning DW, O'Driscoll BR, Powell G, Chew F, Atherton GT, Vyas A, Miles J, Morris J, Niven RM (2009). Randomized controlled trial of oral antifungal treatment for severe asthma with fungal sensitization: The Fungal Asthma Sensitization Trial (FAST) study. Am J Respir Crit Care Med.

[36] Barnes CS, Kennedy K, Gard L, Forrest E, Johnson L, Pacheco F, Hu F, Amado M, Portnoy JM (2008). The impact of home cleaning on quality of life for homes with asthmatic children. Allergy Asthma Proc.

[37] Burr ML, Matthews IP, Arthur RA, Watson HL, Gregory CJ, Dunstan FD, Palmer SR (2007). Effects on patients with asthma of eradicating visible indoor mould: a randomised controlled trial. Thorax.

[38] Katayama N, Fujimura M, Yasui M, Ogawa H, Nakao S (2008). Hypersensitivity pneumonitis and bronchial asthma attacks caused by environmental fungi. Allergol Int.

[39] Iossifova YY, Reponen T, Ryan PH, Levin L, Bernstein DI, Lockey JE, Hershey GK, Villareal M, LeMasters G (2009). Mold exposure during infancy as a predictor of potential asthma development. Ann Allergy Asthma Immunol.

[40] Fairs A, Agbetile J, Bourne M, Hargadon B, Monteiro WR, Morley JP, Edwards RE, Wardlaw AJ, Pashley CH (2013). Isolation of *Aspergillus fumigatus* from sputum is associated with elevated airborne levels in homes of patients with asthma. Indoor Air.

[41] Vesper S, Wymer L (2016). The relationship between environmental relative moldiness index values and asthma. Int J Hyg Environ Health.

[42] Lambrecht BN, Hammad H (2015). The immunology of asthma. Nat Immunol.

[43] Costabel U, Miyazaki Y, Pardo A, Koschel D, Bonella F, Spagnolo P, Guzman J, Ryerson CJ, Selman M (2020). Hypersensitivity pneumonitis. Nat Rev Dis Primers.

[44] Sahin H, Kaproth-Joslin K, Hobbs SK (2019). Hypersensitivity pneumonitis. Semin Roentgenol.

[45] Spagnolo P, Rossi G, Cavazza A, Bonifazi M, Paladini I, Bonella F, Sverzellati N, Costabel U (2015). Hypersensitivity pneumonitis: a comprehensive review. J Investig Allergol Clin Immunol.

[46] Hasan SA, Eksteen B, Reid D, Paine HV, Alansary A, Johannson K, Gwozd C, Goring KA, Vo T, Proud D, Kelly MM (2013). Role of IL-17A and neutrophils in fibrosis in experimental hypersensitivity pneumonitis. J Allergy Clin Immunol.

[47] Blanchet MR, Gold MJ, McNagny KM (2012). Mouse models to evaluate the function of genes associated with allergic airway disease. Curr Opin Allergy Clin Immunol.

[48] Nance S, Cross R, Yi AK, Fitzpatrick EA (2005). IFN-gamma production by innate immune cells is sufficient for development of hypersensitivity pneumonitis. Eur J Immunol.

[49] Abdelsamed HA, Desai M, Nance SC, Fitzpatrick EA (2011). T-bet controls severity of hypersensitivity pneumonitis. J Inflamm (Lond).

[50] Bernatchez E, Langlois A, Brassard J, Flamand N, Marsolais D, Blanchet MR (2017). Hypersensitivity pneumonitis onset and severity is regulated by CD103 dendritic cell expression. PLoS ONE.

[51] Andrews K, Ghosh MC, Schwingshackl A, Rapalo G, Luellen C, Waters CM, Fitzpatrick EA (2016). Chronic hypersensitivity pneumonitis caused by *Saccharopolyspora rectivirgula* is not associated with a switch to a Th2 response. Am J Physiol Lung Cell Mol Physiol.

[52] Nogueira R, Melo N, Novais EBH, Martins N, Delgado L, Morais A (2019). P CM: Hypersensitivity pneumonitis: antigen diversity and disease implications. Pulmonology.

[53] Aimanianda V, Bayry J, Bozza S, Kniemeyer O, Perruccio K, Elluru SR, Clavaud C, Paris S, Brakhage AA, Kaveri SV (2009). Surface hydrophobin prevents immune recognition of airborne fungal spores. Nature.

[54] Riario Sforza GG, Marinou A (2017). Hypersensitivity pneumonitis: a complex lung disease. Clin Mol Allergy.

[55] Selman M, Pardo A, King TE (2012). Hypersensitivity pneumonitis: insights in diagnosis and pathobiology. Am J Respir Crit Care Med.

[56] Thorne PS, Adamcakova-Dodd A, Kelly KM, O'Neill ME, Duchaine C (2006). Metalworking fluid with mycobacteria and endotoxin induces hypersensitivity pneumonitis in mice. Am J Respir Crit Care Med.

[57] Dakhama A, Hegele RG, Laflamme G, Israel-Assayag E, Cormier Y (1999). Common respiratory viruses in lower airways of patients with acute hypersensitivity pneumonitis. Am J Respir Crit Care Med.

[58] Cormier Y, Israel-Assayag E, Fournier M, Tremblay GM (1993). Modulation of experimental hypersensitivity pneumonitis by Sendai virus. J Lab Clin Med.

[59] Gudmundsson G, Monick MM, Hunninghake GW (1999). Viral infection modulates expression of hypersensitivity pneumonitis. J Immunol.

[60] Ohshimo S, Guzman J, Costabel U, Bonella F (2017). Differential diagnosis of granulomatous lung disease: clues and pitfalls. Eur Respir Rev.

