# Supplementary figures and images for: Novel acute hypersensitivity pneumonitis model induced by airway mycosis and high dose lipopolysaccharide
Source: Respir Res. 2021 Oct 10;22:263. doi: 10.1186/s12931-021-01850-5 (PMC8503997; doi:10.1186/s12931-021-01850-5)

Supplementary Figure 1.

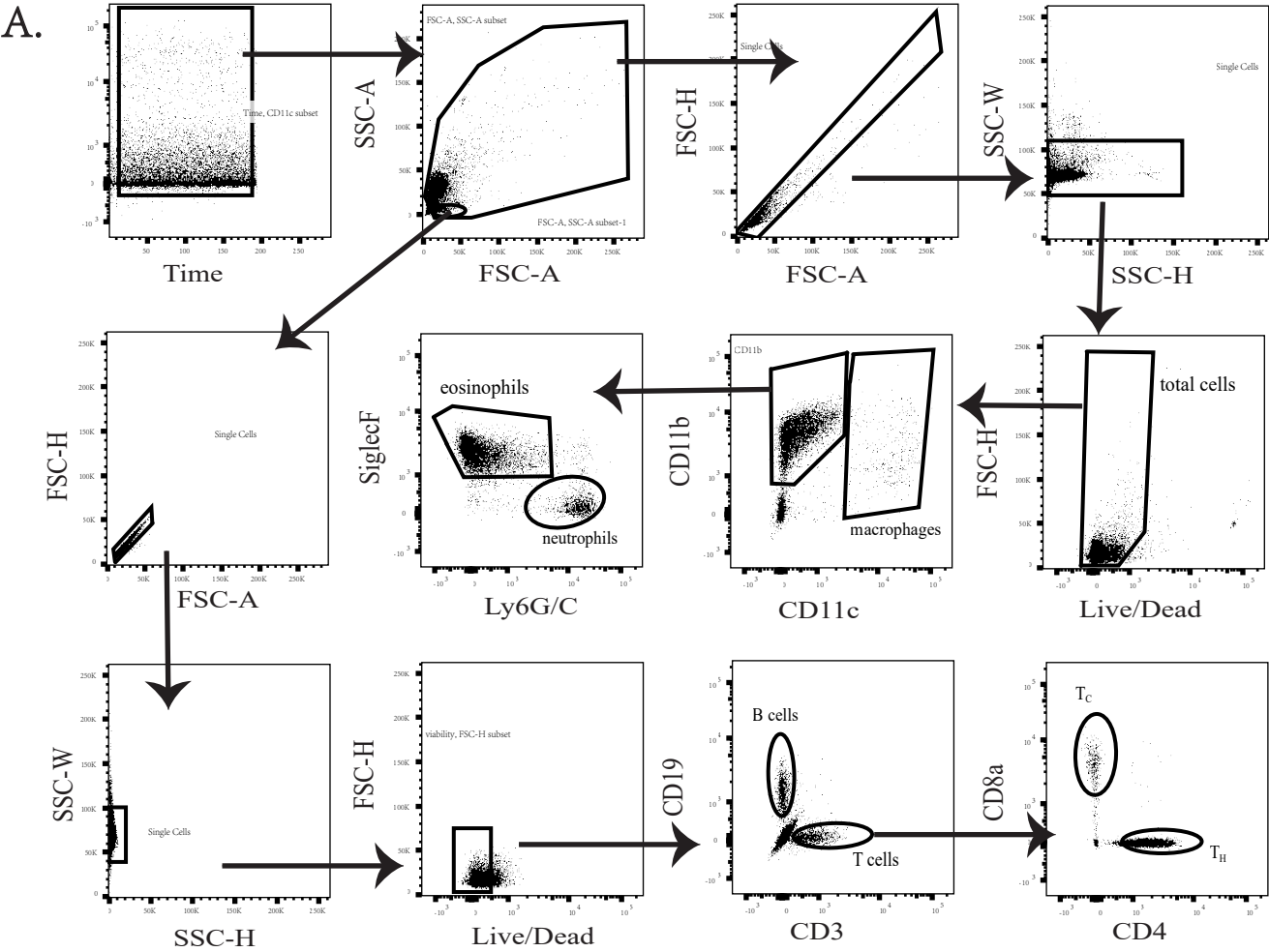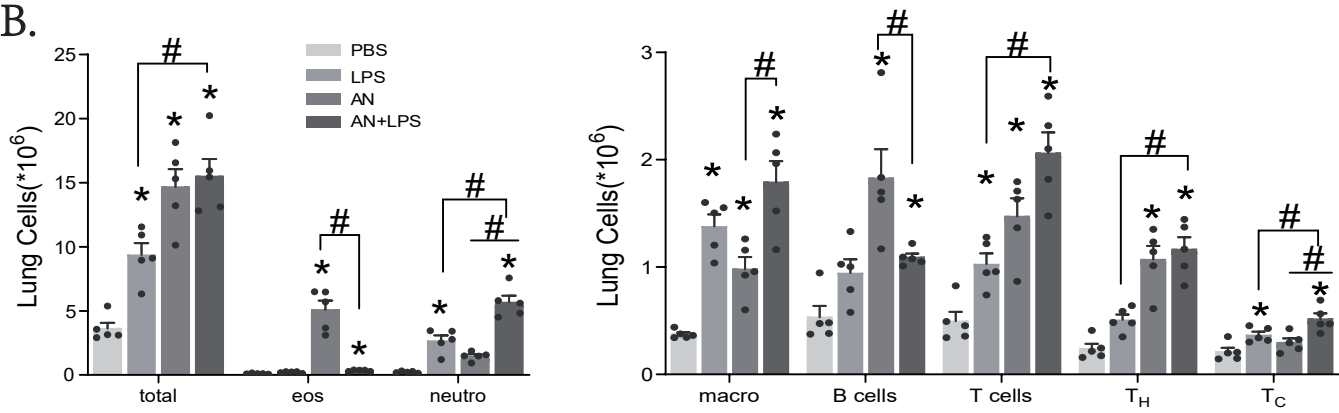

Supplement: Supplementary file 1 — Additional file 1: Fig. 1: The effect of LPS on the composition of inflammatory cells in the lungs. (A) Flow cytometric analytical scheme of the differential of BALF cells and lung cells. (B) Lung cells were analyzed for inflammatory cell numbers. Results are presented as the mean ± SEM (n=5 in each group). *p<0.05 compared with PBS administration; #p<0.05 between indicated groups. Data are from one experiment. [file 12931_2021_1850_MOESM1_ESM.pdf]
