# Supplementary material for: Novel acute hypersensitivity pneumonitis model induced by airway mycosis and high dose lipopolysaccharide
Source: Respir Res. 2021 Oct 10;22:263. doi: 10.1186/s12931-021-01850-5 (PMC8503997; doi:10.1186/s12931-021-01850-5)
Supplement: Supplementary file 2 — Additional file 2: Table S1. mouse antibody marker, fluorphore, clone and company used for cytometric analysis of lung cells. [file 12931_2021_1850_MOESM2_ESM.docx]

Supplementary Table 1

| Marker | Color | Clone | Company |
| --- | --- | --- | --- |
| CD16/32 | —— | clone 93 | Biolegend |
| CD11b | PE | clone M1/70 | Invitrogen |
| CD11c | APC | clone HL3 | BD Pharmingen |
| Siglec-F | PerCP-Cy5.5 | clone E50-2440 | BD Pharminge |
| Ly6G/C | APC-Cy7 | clone HL3 | BD Pharmingen |
| CD3 | eFluor450 | clone 17A2 | ThermoFisher |
| CD19 | BV510 | clone 6D5 | Biolegend |
| CD4 | BV785 | clone RM4-5 | Biolegend |
| CD8a | BV711 | clone 53-6.7 | Biolegend |
| Viability | Live/dead Fixable Blue | —— | ThermoFisher |
| CD4 | APC | clone RM4-5 | Biolegend |
| CD3ε | APC-Cy7 | clone 145-2C11 | Biolegend |
| IFN-γ | BV750 | clone XMG1.2 | BD Bioscience |
| IL-5 | PE | clone TRFK5 | BD Pharmingen |
| IL-13 | Alexa Fluor 488 | clone eBio13A | ThermoFisher |
| IL-17A | BV650 | clone TC11-18H10.1 | Biolegend |

Supplementary Table 1: Table showing the mouse antibody marker, fluorphore, clone and company used for cytometric analysis of lung cells.
