# Supplementary material for: Novel acute hypersensitivity pneumonitis model induced by airway mycosis and high dose lipopolysaccharide
Source: Respir Res. 2021 Oct 10;22:263. doi: 10.1186/s12931-021-01850-5 (PMC8503997; doi:10.1186/s12931-021-01850-5)
Supplement: Supplementary file 3 — Additional file 3: Table S2. Histopathologic description and score of mouse lungs by group.. [file 12931_2021_1850_MOESM3_ESM.docx]

Supplementary Table 2

| Treatment Group | General Pathologic Description  (hematoxylin and eosin staining) | # Granulomas, whole lung (mean ± SD) |
| --- | --- | --- |
| PBS | No to minimal inflammation consisting of scant peribronchial monocytosis | 0 |
| LPS 1ug | Prominent interstitial and peribronchovascular inflammation consisting of primarily mononuclear cells and few neutrophils | 0 |
| AN 400k | Diffuse peribronchovascular and alveolar, predominantly eosinophilic inflammation with numerous scattered multinucleate giant cells | 1.8 ±1.6 |
| AN + LPS | Predominantly peribronchovascular and interstitial mononuclear inflammation with few neutrophils with rare multinucleate giant cells | 9.6 ± 3.2 |
